# Linkage to hepatitis C care after incarceration in jail: a prospective, single arm clinical trial

**DOI:** 10.1186/s12879-019-4344-1

**Published:** 2019-08-08

**Authors:** Matthew J. Akiyama, Devin Columbus, Ross MacDonald, Alison O. Jordan, Jessie Schwartz, Alain H. Litwin, Benjamin Eckhardt, Ellie Carmody

**Affiliations:** 1 Montefiore Medical Center/Albert Einstein College of Medicine, 3300 Kossuth Ave, Bronx, NY 10467 USA; 2 New York University School of Medicine, New York, NY USA; 3 New York City Health + Hospitals, Correctional Health Services, New York, NY USA; 4 New York City Department of Health and Mental Hygiene, Queens, NY USA; 5 University of South Carolina School of Medicine, Greenville, South Carolina USA; 6 Clemson University School of Health Research, Clemson, South Carolina USA; 7 Prisma Health - Upstate, Greenville, South Carolina USA

**Keywords:** HCV, Jail, Prison, Linkage to care, PWID

## Abstract

**Background:**

Hepatitis C virus (HCV) is a major public health problem in correctional settings. HCV treatment is often not possible in U.S. jails due to short lengths of stay. Linkage to care is crucial in these settings, but competing priorities complicate community healthcare engagement and retention after incarceration.

**Methods:**

We conducted a single arm clinical trial of a combined transitional care coordination (TCC) and patient navigation intervention and assessed the linkage rate and factors associated with linkage to HCV care after incarceration.

**Results:**

During the intervention, 84 participants returned to the community after their index incarceration. Most participants were male and Hispanic, with a history of mental illness and a mean age of 45 years. Of those who returned to the community, 26 (31%) linked to HCV care within a median of 20.5 days; 17 (20%) initiated HCV treatment, 15 (18%) completed treatment, 9 (11%) had a follow-up lab drawn to confirm sustained virologic response (SVR), and 7 (8%) had a documented SVR. Among those with follow-up labs the known SVR rate was (7/9) 78%. Expressing a preference to be linked to the participant’s existing health system, being on methadone prior to incarceration, and feeling that family or a loved one were concerned about the participant’s wellbeing were associated with linkage to HCV care. Reporting drinking alcohol to intoxication prior to incarceration was negatively associated with linkage to HCV care.

**Conclusion:**

We demonstrate that an integrated strategy with combined TCC and patient navigation may be effective in achieving timely linkage to HCV care. Additional multicomponent interventions aimed at treatment of substance use disorders and increasing social support could lead to further improvement.

**Trial registration:**

Clinicaltrials.gov NCT04036760 July 30th, 2019 (retrospectively registered).

## Background

As many as half a million people in correctional settings in the United States may be hepatitis C virus (HCV) antibody-positive [1]. Due in part to the criminalization of individuals with substance use disorders known to increase HCV risk, HCV prevalence in correctional settings is in the order of 10–20 times that of the general population [2]. It is estimated that one in three people living with HCV (PLWHCV) pass through the criminal justice system each year [3]. Failure to effectively intervene for PLWHCV in HCV care within the criminal justice system may place PLWHCV at ongoing risk for progression of HCV-related liver disease (cirrhosis, end-stage liver disease, and hepatocellular carcinoma) and may facilitate ongoing transmission of HCV in the neighborhoods of greatest socio-economic disparities to which PLWHCV return after incarceration [4–6].

The criminal justice system, being a high prevalence setting, is an important locus for HCV treatment with the goal of HCV elimination [7]. For optimal operationalization of a response to HCV in this setting it is important to differentiate jails from prisons. People incarcerated in local jails are largely detained pre-trial and/or have short sentences of generally up to one year or less. People incarcerated in prisons are convicted of a crime and typically sentenced to incarceration for more than one year. While longer sentences more readily permit HCV treatment in prisons, treatment is complicated in jails where the median length of stay is 15 days [8]. HCV regimens have been reduced to twelve weeks or less for the majority of individuals with direct acting antiviral (DAA) therapy making treatment feasible for some jail detainees [9]. However, there are twelve million admissions to US jails per year, which is nineteen times the number in state and federal prisons [10]. With the high volume of likely untreated detainees cycling through, U.S. jails are a critical site for linkage to HCV care after incarceration.

Linkage to community care from correctional settings is complicated by competing priorities after incarceration. During incarceration, individuals experience social, structural, and medical disruptions that complicate the reentry period. To date, the majority of work on linkage to care from correctional settings has focused on people living with HIV (PLWHIV). Lack of access to health insurance, lack of social support, exacerbation of mental health conditions, and unstable housing have all been documented as complicating linkage to care among PLWHIV [11–14].

A variety of strategies have been evaluated for their impact on linkage to care among PLWHIV leaving jail and prison. Case management, which aims to coordinate medical, mental health, social, and other services has been the most extensively studied. Evidence suggests that case management plays a key role in facilitating linkage to HIV care. In one randomized controlled trial (RCT), case management was shown to be more effective than referral alone [15]. Case management has also been associated with improved adherence to antiretroviral therapy (ART), HIV viral load suppression, retention in care, fewer emergency department visits, and less housing instability and food insecurity among PLWHIV [16, 17]. Transitional care coordination (TCC) is a form of case management that includes an intake assessment, discharge and transitional care plans, and appointment scheduling. TCC is now a nationally recognized evidence-informed intervention that plays a key role in facilitating linkage to HIV care after incarceration [18]. Patient navigation is another strategy that may provide additional benefit. While patient navigation does not have a standard definition, programs incorporating this strategy tend to focus on advocacy, health education, and social support [19]. In a recently published RCT study, patient navigation coupled with case management resulted in greater linkage to and retention in HIV care after incarceration in jail than standard case management alone [20].

Strategies for linkage to HIV care may be applicable to other clinical and therapeutic areas including HCV. To date, models of care and their impact on linkage to HCV care after incarceration are poorly understood. There are also few data on predictors of linkage to HCV care after incarceration. We conducted a prospective, single arm clinical trial of a combined TCC and patient navigation intervention among HCV mono-infected individuals leaving the New York City jail system. The aim of this study was to measure linkage to and retention in care associated with this combined TCC and patient navigation model on the rate of linkage to and retention in HCV care. We also aimed to identify factors associated with linkage to HCV care and evaluate feasibility of operationalizing this care coordination strategy among justice-involved PLWHCV.

## Methods

### Study setting and participants

The NYC jail system is the second largest in the U.S. with nearly fifty thousand admissions per year and an average daily census of over eight thousand (as of 2018) [21]. Health care service delivery in jails is the responsibility of Correctional Health Services, a division of NYC Health + Hospitals, the nation’s largest public health care system [21]. We identified potential participants using clinical data reports identifying individuals with detectable HCV viral loads. Recruitment was conducted in three NYC jails and one hospital correctional health ward (Bellevue Hospital). We included consenting patients who were ≥ 18 years old at time of recruitment, were diagnosed with chronic HCV, and had a projected length of stay sufficient to anticipate community reentry within the study follow-up period. Exclusion criteria included HIV (due to existing TCC services on a population basis) and inability to obtain informed, signed consent. We conducted study enrollment and baseline interviews in private cubicles of jail health clinics.

### Participant tracking

We tracked dates of incarceration and community return by querying public inmate lookup databases [22, 23] and communicating with jail health staff throughout the course of the study. Index incarceration was defined as the incarceration during which study enrollment and baseline interview took place. Reincarceration was defined as any incarceration of duration of greater than 24 h occurring after the index incarceration.

We collected detailed contact information for participants and participants’ next of kin to facilitate patient navigation upon reentry into the community. We also collected information on places and programs the participant may have frequented in the community (e.g., drug treatment centers; homeless shelters).

### HCV transitional care coordination & patient navigation

We partnered with New York City Correctional Health Services teams that employ the well-established TCC intervention to facilitate linkages to care for PLWHIV after incarceration in the NYC jail system [18]. The TCC intervention includes: intake and assessment services, discharge planning, health insurance assistance, court liaison to facilitate medical alternatives to incarceration, facilitating continuity of medication and patient navigation and coordination with service providers to facilitate linkages to care after incarceration [12]. We developed a care coordination intervention employing a similar model for HCV mono-infected PLHCV with HCV education and community patient navigation performed by a master’s level study coordinator. Patient navigation included reminder calls, appointment rescheduling if needed, appointment accompaniment if requested, and free public transit passes for each successfully completed HCV appointment. Patient navigation did not include home visits or direct outreach due to resource limitations. We gave all participants contact information for key study team members and encouraged communication before and after return to the community.

Study personnel offered participants assistance with scheduling HCV linkage appointments at their existing health system, if applicable, or one of two study-affiliated community clinical partners if they preferred. Appointment dates were targeted within two to four weeks of community return. Whether or not a community appointment was scheduled during the jail stay, we attempted to engage all participants within one week after incarceration using information from the enrollment interview. This included attempting to contact participants and/or next of kin via telephone, text, and/or email. Participants were followed for up to 180 days after the index incarceration during which time attempts were made at linkage to HCV care.

For participants contacted after incarceration, we made up to five attempts to schedule community HCV appointments within 180 days after the index incarceration before categorizing a participant as not linked. If the participant was reincarcerated prior to or after being linked to HCV care, we attempted to coordinate follow-up appointments after reincarceration. For participants who linked to care, study personnel assisted with scheduling HCV clinical and laboratory follow-up visits.

### Data sources and covariates

Following enrollment, we conducted participant interviews using a modified version of the Addiction Severity Index (ASI) to identify potential factors associated with linkage to HCV care. The ASI includes information on medical history, employment/financial support, drug/alcohol use (e.g., Have you used heroin in your lifetime; how many days did you use heroin in the 30 days prior to incarceration?), legal case status, family/social relationships, and psychiatric status. Individual participants’ answers were recorded by a study coordinator and entered into REDCap – a secure, web-based application designed to support data capture for research studies [24]. Feasibility data were gathered from study staff logs detailing the number of attempted and successful contacts with participants/next of kins. Data on linkage to and retention in HCV care were obtained from participant medical records.

### Primary and secondary outcomes

Our primary outcome was linkage to HCV care, which was defined as a documented visit with an HCV treating provider within 180 days after the index incarceration. Once linked to care, clinicians assessed participants for HCV treatment eligibility according to standard of care. Secondary outcomes for treatment-eligible participants included treatment initiation, treatment completion, and documented sustained virologic response (SVR), or HCV cure. For those who linked to care we continued to assess for secondary outcomes beyond 180 days if applicable.

### Statistical analysis

We conducted a bivariate analysis to examine associations with linkage to HCV care using Chi-square and Fisher’s exact tests for categorical variables. Significance was assessed at an alpha level of 0.05. Statistical analyses were conducted using SAS 9.2 (SAS Institute, Cary, NC). We also used EventFlow (University of Maryland, Human-Computer Interaction Lab; http://hcil.umd.edu/eventflow) to descriptively identify relevant temporal event sequences associated with the HCV corrections-community care cascade with an emphasis on linkage to HCV care and reincarceration.

### Statement of ethical standards

The study team obtained written informed consent from all participants enrolled in the study, and approval to conduct the study was obtained from New York University School of Medicine and New York City Department of Health and Mental Hygiene Institutional Review Boards (IRB). The New York University School of Medicine IRB approval process encompassed detailed review by the IRB prisoners’ advocate.

## Results

### Participants

**Fig. 1:**
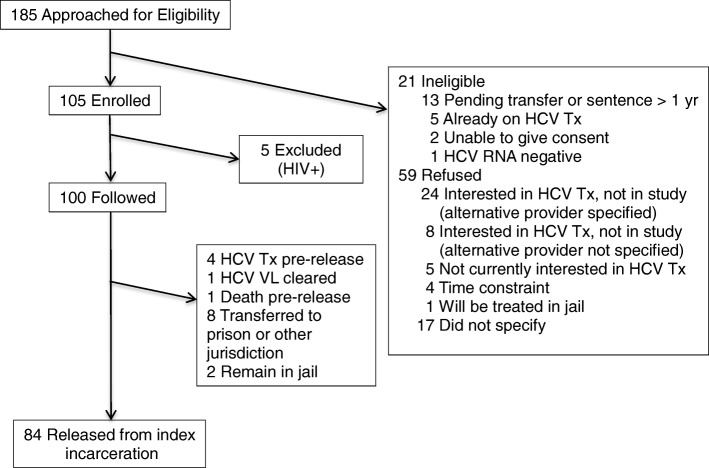
Participant flow chart. HCV = hepatitis C virus, HIV = human immunodeficiency virus, VL = viral load, Tx = treatment

From May 2015 to April 2017 we identified and interviewed 185 people incarcerated in NYC jails for study participation; a total of 105 were enrolled and 5 were subsequently disenrolled as PLWHIV from the study group, leaving a cohort of 100 who were followed during and after their index incarceration. Of the 100 participants, 8 were transferred to state prison or another jurisdiction, 4 received HCV treatment in jail, 1 spontaneously cleared their HCV infection, 1 died prior to release, and 2 remained incarcerated at the completion of the study; leaving 84 who returned to the community after the index incarceration (Fig. 1).

Most participants were male (57%) and Hispanic (55%) with a mean age of 45 years (standard deviation, SD = 12). The majority (63%) had a reported history of mental illness. Over one quarter (26%) reported being homeless prior to incarceration. Most (81%) participants reported having health insurance in the community, but fewer than one quarter (24%) had an existing primary care provider and fewer than 10% had an existing HCV provider. Over two thirds (70%) reported a lifetime history of injection drug use and nearly two thirds (61%) reported injection drug use in the 30 days prior to incarceration. Most (89%) reported using heroin in their lifetime and over half reporting heroin use in the 30 days prior to incarceration. Nearly two thirds (60%) reported taking methadone prior to incarceration and nearly one third (29%) reported taking buprenorphine-naloxone (Table 1).

**Table 1:** Participant characteristics and associations with linkage to HCV care

Characteristics	Linked to care *N* = 26* (%)	Unlinked to care *N* = 58* (%)	Total *N* = 84* (%)	*P* value
Age, mean (SD)	47 (11)	44 (12)	45 (12)	0.29
≥ 45	14 (54)	29 (50)	43 (51)	0.74
< 45	12 (46)	29 (50)	41 (49)	
Sex				0.31
Female	9 (35)	27 (47)	36 (43)	
Male	17 (65)	31 (53)	48 (57)	
Race/ethnicity				0.94
Hispanic	15 (58)	31 (54)	46 (55)	
NH Black	5 (19)	10 (18)	15 (18)	
NH White	5 (19)	12 (21)	17 (21)	
Other	1 (4)	4 (7)	4 (6)	
Health insurance^b^	22/26 (85)	45/57 (79)	67/83 (81)	0.54
Homeless^b^	5 (19)	17 (29)	22 (26)	0.11
Graduated high school	14/25 (56)	34/55 (62)	48/80 (60)	0.62
Psychiatric diagnosis^a^	13 (50)	40 (69)	53 (63)	0.10
Care Coordination				
Personal contact info at enrollment	13 (50)	23 (40)	36 (43)	0.38
Next of kin contact info at enrollment	25 (96)	52 (90)	77 (92)	0.32
Known release date	14 (54)	34 (59)	48 (57)	0.68
HCV appointment scheduled pre-release	13 (50)	19 (33)	32 (38)	0.13
Existing HCV provider	3/24 (13)	3/54 (6)	6/78 (8)	0.29
Existing primary care provider	9/24 (38)	10/55 (18)	19/79 (24)	0.06
Prefer linkage to existing health system	8 (31)	7 (12)	15 (18)	0.04**
Opioid agonist therapy^b^				
Methadone	20 (77)	30 (52)	50 (60)	0.03**
Suboxone	5 (19)	19 (33)	24 (29)	0.21
Substance use				
Injection drug use				
30 days prior	16 (62)	35 (60)	51 (61)	0.92
Lifetime	22 (85)	36 (63)	58 (70)	0.22
Heroin				
30 days prior	13 (50)	34 (59)	47 (56)	0.46
Lifetime	25 (96)	50 (86)	75 (89)	0.17
Prescription opiates				
30 days prior	4 (15)	2 (3)	6 (7)	0.90
Lifetime	14 (54)	23 (40)	37 (44)	0.22
Crack/Cocaine				
30 days prior	9 (35)	23 (40)	32 (38)	0.66
Lifetime	21 (81)	48 (83)	69 (82)	0.83
Amphetamines				
30 days prior	0 (0)	2 (3)	2 (2)	0.33
Lifetime	2 (8)	8 (14)	10 (12)	0.42
Marijuana				
30 days prior	6 (23)	20 (34)	26 (31)	0.30
Lifetime	15 (58)	44 (76)	59 (70)	0.09
Alcohol to intoxication				
30 days prior	2 (8)	12 (21)	14 (17)	0.14
Lifetime	5 (16)	26 (44)	31 (37)	0.03**
Social support				
Feel supported socially	21/25 (84)	37/56 (66)	58/81 (72)	0.10
Feel family or a loved one is concerned about wellbeing	25/25 (100)	45/56 (64)	70/81 (86)	0.02**
Reincarcerated ≥ 1 time within 180 days post-release	8 (31)	20 (36)	28 (33)	0.86

Data were obtained at enrollment in the jail setting. ^a^ Includes depression, anxiety, bipolar, schizophrenia; ^b^Refers to the period prior to incarceration; *Unless denominator specified; **Statistically significant alpha level *p* < 0.05. *HCV* hepatitis C virus, *NH* non-Hispanic, *SD* standard deviation

### HCV transitional care coordination & patient navigation

The median duration of time between enrollment and community return after the index incarceration was 34.0 days (interquartile range, IQR = 71.8). Less than one half (43%) provided personal contact information, but almost all provided contact information for a next of kin (92%). On average, participants were engaged 1.2 (SD = 0.5) times by study staff during incarceration. In the 180 days after incarceration, an average of 3.8 (SD = 4.4) contact attempts were made resulting in 42 (50%) participants being successfully contacted, and an average of 2.7 (SD = 2.1) attempts to contact next of kin resulted in 53 (63%) successful contacts of participants’ next of kin.

### Linkage to & retention in HCV care

**Fig. 2:**
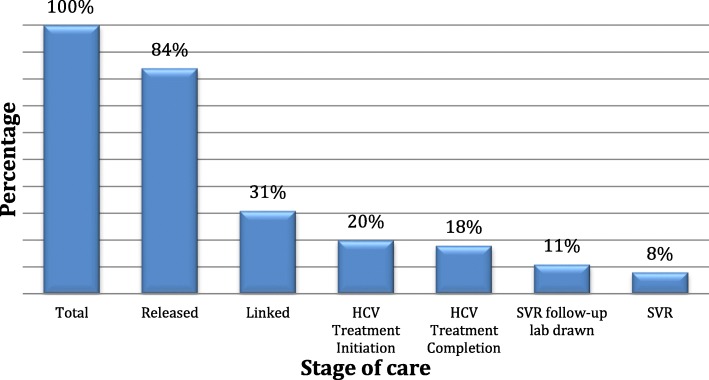
Hepatitis C virus (HCV) care cascade after incarceration in the New York City jails. In a cohort of 84 participants who returned to the community after incarceration, 26 (31%) linked to HCV care, 17 (20%) initiated HCV treatment, 15 (18%) completed treatment, 9 (11%) had a follow-up lab drawn to confirm sustained virologic response (SVR), and 7 (8%) had a documented SVR. Of the 9 participants with documented 12-week follow-up labs, this was a 78% SVR rate

Of the participants who returned to the community after the index incarceration, 26 (31%) linked to HCV care within a median of 20.5 (IQR = 69.8) days. On average, 1.1 (SD = 1.1) linkage appointments were made among all participants who returned to the community. In terms of secondary outcomes, 17 (20%) participants initiated HCV treatment, 15 (18%) completed treatment, 9 (11%) had a follow-up lab drawn to confirm SVR, and 7 (8%) had a documented SVR (Fig. 2). Therefore, the SVR rate among those with follow-up was (7/9) 78%. One participant who linked to care and initiated HCV treatment was reincarcerated mid-treatment. Our study coordinator received a phone call from a jail physician to confirm the participant was on treatment. The participant was continued on treatment and achieved SVR in jail.

### Reincarceration and loss to follow-up

**Fig. 3:**
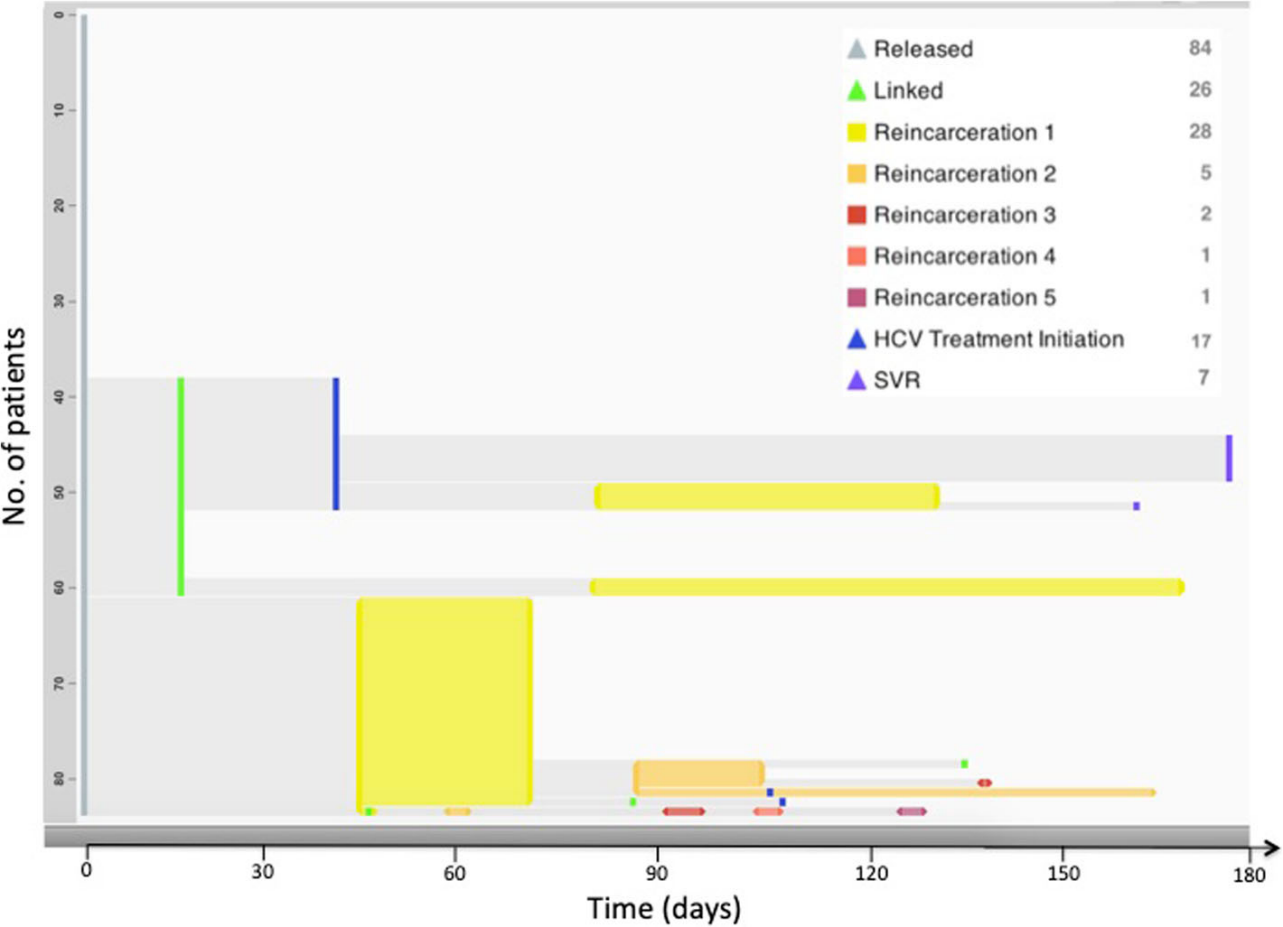
Trajectories of people living with HCV after incarceration in the New York City Jails. Each row in an EventFlow figure represents one participant’s sequence of events during a period of time. The height of each bar is proportional to the number of records with that sequence, and its horizontal position is determined by the median time between events. Groups of sequences with the same preceding event are sorted by the number of records in each group. The sequence groups are shown from top to bottom in descending order of number of participants per group. For interval events such as returns to jail, the length of the intervals represents the mean duration of the grouped events. For point events such as linkage to HCV care, periods between the aggregated point events represent the mean length of time from any previous point event

Overall, 28 (33%) participants were reincarcerated within a median of 88.0 (IQR = 85.8) days after their index incarceration. Reincarceration was an equally common initial event compared with linkage to HCV care with 23 (27%) participants being reincarcerated following community return. Five participants who were linked to HCV care were subsequently reincarcerated. Conversely, three participants who were reincarcerated subsequently linked to HCV care after incarceration. Nearly half (45%) of study participants were either not known to be linked to HCV care or reincarcerated in the 180 days after their index incarceration (Fig. 3).

### Associations with linkage to HCV care

Expressing a preference to be linked to the participant’s existing health system, being on methadone prior to incarceration, and feeling that family or a loved one were concerned about the participant’s wellbeing were associated with linkage to HCV care. Reporting drinking alcohol to intoxication prior to incarceration was negatively associated with linkage to HCV care. Notably, homelessness was not significantly associated with linkage to HCV care and the proportion of those reincarcerated was similar in the group who were linked to care (8/26, 31%) and the group who were not linked to care (20/58, 36%, Chi-square *p* = 0.86) (Table 1).

## Discussion

To our knowledge, this is the first prospective trial to characterize the HCV care cascade in the transition from a correctional setting to the community. It is also the first cohort study to describe factors associated with linkage to HCV care after incarceration. These data provide preliminary evidence that a combined TCC and patient navigation strategy may be effective in achieving timely linkage to HCV care for a subset of PLWHCV after incarceration in jail.

Linkage rates in our study are similar to those reported in other corrections-based public health programs and pilot studies from other jurisdictions. In a Rhode Island-based rapid HCV testing pilot study in which participants received a questionnaire, informational video, and a referral, 4/15 (27%) participants with confirmed viremia linked to HCV care [25]. In a Massachusetts program in which participants with HCV received referrals to HCV care, 14/82 (17%) and 31/82 (38%) had laboratory evidence of linkage to HCV care within 6 and 12 months, respectively [26]. In an HCV testing and linkage to care program in South and North Carolina, participants received HCV post-test counseling, a referral, appointment scheduling, and patient navigation (in North Carolina only). In South and North Carolina, respectively, 2/7 (29%) and 10/18 (56%) participants who were referred attended their first HCV appointment [27].

All of these studies suggest there are substantial challenges associated with linkage to HCV care. Moreover, data from South Carolina, Massachusetts, and Rhode Island demonstrate these barriers are not overcome with education, referral, and appointment scheduling alone. While data from North Carolina suggest better linkage rates may be achievable with patient navigation, the small sample size limits the generalizability of the results and, still, only roughly half of participants linked to HCV care. Our intervention was modeled on the evidence-informed TCC intervention used in NYC jails to facilitate linkage to HIV care after incarceration. For PLWHIV, linkage rates associated with this program approach 75% [18]. Yet, the linkage rate was substantially lower among monoinfected PLWHCV in our study.

Several factors may account for the higher linkage rate among PLWHIV than PLWHCV. First, in our study we used telephone-based patient navigation with appointment accompaniment if requested as the community portion of the intervention. The TCC intervention for PLWHIV after incarceration in the NYC jails includes an extensive network of community resources, linkage agreements with community providers, dedicated community case managers funded through Ryan White Part A, and housing assistance [28]. We were not able to provide such extensive services due to resource limitations and believe such services could have improved linkage rates [18].

We hypothesize there are several other key differences leading to higher linkage rates among PLWHIV than PLWHCV. First, due to the earlier response of health care systems to the HIV epidemic at the federal, state, and local levels and relatively longstanding availability of effective ART, many PLWHIV are more informed about their HIV diagnoses and the effectiveness of ART. As an example, Loeliger et al. demonstrated that an HIV diagnosis of greater than one year predicted retention in HIV care among justice-involved PLWHIV [17]. PLWHCV may be relatively unaware of the short- and long-term consequence of HCV infection and the effectiveness of DAA therapy. With increased collective HCV- and DAA-related knowledge, linkage rates may marginally improve over time.

Additionally, relationships with medical providers prior to incarceration have been shown to result in higher linkage rates among PLWHIV [16, 29, 30]. Our study mirrors this finding given a preference to be linked to one’s existing health system was associated with linkage to HCV care. Among PLWHIV who reported no prior history of HIV medical services, Molitor et al. identified a linkage rate of 29% [31]. Very few of our study participants reported an existing relationship with an HCV provider prior to incarceration, and our linkage rate was similar. Taken together, these findings suggest increased familiarity with the healthcare system may facilitate linkage to care.

Active substance use disorders also likely contribute to lower linkage rates among PLWHCV. Injection drug use is the number one risk factor for HCV in the U.S., and nearly two-thirds of our study participants reported injection drug use thirty days prior to incarceration. Substance use disorders are known to complicate linkage among PLWHIV [32]. In our study, injection drug use was not associated with linkage to HCV care; however, taking methadone prior to incarceration was. Opioid agonist therapy is an evidence-based strategy leading to more stability from active drug use and higher linkage to care among PLWHIV [20]. While we do not have follow-up data on continuation of methadone, we presume many of these participants continued on methadone as those who were on methadone prior to incarceration at the time of this study were generally maintained in the NYC jails unless they were expected to be transferred to prison based on available legal criteria. Given that the period after incarceration is associated with a high risk for active drug use and theoretical risk for HCV transmission [33–36], rapid linkages to HCV and substance use disorder treatment are a high priority. The importance of linkage interventions among people who inject drugs is further underscored by risk of overdose after incarceration [37].

Reporting a history of drinking alcohol to intoxication prior to incarceration was negatively associated with linkage to HCV care. Data are limited on the impact of alcohol use on linkage to care following incarceration. However, alcohol has been shown to be negatively associated with DAA adherence among people who inject drugs [38]. Drinking alcohol to intoxication in the 30 days prior to incarceration was not associated with linkage to care; however, it did show a similar trend. Therefore, screening and treatment of alcohol use disorders should also be considered an integral component of HCV linkage to care programs.

Feeling family or a loved one were concerned about the participants’ wellbeing was also associated with linkage to HCV care in our study. The role of social support in promoting linkage to care after incarceration among PLWHCV is not known; however, there is evidence among PLWHIV that a lack of social support is a barrier. The use of patient or peer navigators with racial/ethnic concordance or shared life experience have been proposed as an important strategy [31, 39, 40]. This may be even more important for those who lack support from family or a loved one. In our study, a navigator with a master’s level of education performed patient navigation that was mostly telephonic. It is possible peer navigation with active outreach might have resulted in a higher linkage rate.

We did not identify a statistically significant relationship between reincarceration and linkage to HCV care. Reincarceration has been demonstrated to be a complicating factor in linkage to and retention in HIV care. However, since incarcerated persons have access to stable medical services, it can also be leveraged to improve HIV-related outcomes [17, 41, 42]. In our study, reincarceration was more common in the 180 days after index incarceration than linkage to HCV care and was an equally common initial event to linkage to HCV care. If individuals are reincarcerated, communication can be interrupted and HCV linkage appointments may be missed. Conversely, reincarceration may be an opportunity to reengage individuals with HCV who have not yet linked to care, as was the case for three participants in this study. For those who initiate HCV treatment in the community and are at risk of ongoing justice-involvement, education should be provided to make jail healthcare staff aware to avoid HCV treatment interruption.

This study has limitations. First, as a single arm trial, we are unable to determine if the observed linkage rate was directly attributable to the intervention. Moreover, the moderate sample size could lead to decreased statistical power to detect associations. However, we believe our study provides important preliminary data on the rate and factors associated with linkage to HCV care after incarceration in jail following the implementation of a combined case management and patient navigation strategy. Second, the study took place in one large urban area, and community partners were able to schedule HCV appointments within 2–4 weeks after incarceration, which may limit generalizability. Third, our sample may not have been representative of PLWHCV in the jail population as a whole since the median duration of time between enrollment and community return was 34 days, and detainees with the shortest lengths of stay were not able to be recruited. Fourth, demographics and covariates like mental illness were obtained through self-report, which may under- or overestimate true rates. Lastly, follow-up for linkage to HCV care was conducted over 180 days after the index incarceration and participants who were lost to follow-up could have linked to non study-affiliated clinics so our linkage and retention outcomes may be underestimated.

## Conclusions

In conclusion, our study provides important real-world data on the rate and factors associated with linkage to HCV care after incarceration jail. Due to the modest linkage rates observed, future interventions should consider strengthening transitional care planning and community patient navigation among justice-involved PLWHCV. We believe that multicomponent intervention incorporating education, increased resources for TCC and community-based patient navigation, treatment of substance use disorders – specifically concomitant linkage to opioid agonist therapy, and increasing social support are needed.

## Data Availability

The datasets used and/or analysed during the current study are available from the corresponding author on reasonable request.

## Abbreviations

ART: Antiretroviral therapy
ASI: Addiction Severity Index
DAA: Direct-acting antiviral
HCV: Hepatitis C virus
PLWHCV: People living with HCV
PLWHIV: People living with HIV
RCT: Randomized controlled trial
SVR: Sustained virologic response
TCC: Transitional care coordination
